# Targeting Molecular Pathways in Intracranial Metastatic Disease

**DOI:** 10.3389/fonc.2019.00099

**Published:** 2019-03-04

**Authors:** Vyshak Alva Venur, Justine V. Cohen, Priscilla K. Brastianos

**Affiliations:** Divisions of Neuro-Oncology and Hematology/Oncology, Departments of Neurology and Medicine, Massachusetts General Hospital, Harvard Medical School, Boston, MA, United States

**Keywords:** brain metastases (BM), targeted therapy, breast cancer, lung cancer, melanoma

## Abstract

The discovery and clinical application of agents targeting pivotal molecular pathways in malignancies such as lung, breast, renal cell carcinoma, and melanoma have led to impressive improvements in clinical outcomes. Mutations in epidermal growth factor receptor (*EGFR*), and rearrangements of anaplastic lymphoma kinase (*ALK*) are targetable in lung cancer, while *BRAF* mutations have been successfully targeted in metastatic melanoma. Targeting estrogen receptors, cyclin dependent kinases, and *HER2* (Human Epidermal Receptor) have resulted in improvement in survival in breast cancer. Major strides have been made in the management of metastatic renal cell carcinoma by targeting the vascular endothelial growth factor (VEGF) pathway. However, intracranial metastases remain a major hurdle in the setting of targeted therapies. Traditional treatment options for brain metastases include surgery, whole brain radiation therapy (WBRT), and stereotactic radiosurgery (SRS). Surgery is effective in symptomatic patients with dominant lesions or solitary intracranial metastases, however, recovery time can be prolonged, often requiring an interruption in systemic treatment. WBRT and SRS provide symptomatic relief and local control but data on improving overall survival is limited. Most targeted therapies which provide extracranial control have limited penetration through the blood brain barrier. Given the limited therapeutic options and increasing prevalence of brain metastases, finding new strategies for the management of intracranial metastatic disease is critical. Genomic analysis of brain metastases has led to a better understanding of variations in the driver mutations compared to the primary malignancy. Furthermore, newer generations of targeted agents have shown promising intracranial activity. In this review, we will discuss the major molecular alterations in brain metastases from melanoma, lung, breast, and renal cell carcinoma. We will provide an in-depth review of the completed and ongoing clinical trials of drugs targeting the molecular pathways enriched in brain metastases.

## Introduction

Brain metastases, a common manifestation of advanced solid malignancies, are associated with significant morbidity and mortality. The incidence of brain metastases varies with primary tumor type, and the overall estimate of the incidence is unclear. Lung cancer is the most common cause of brain metastases; small cell lung cancer contributes to up to 50% of brain metastases from lung cancer (1). Breast cancer is the second most common cause of brain metastases; about half of all brain metastases in breast cancer patients occur in *HER2* (human epidermal growth factor-2) overexpressing breast cancer, followed by triple negative breast cancer, and hormone receptor positive breast cancer (2). The highest frequency of brain metastases is seen in patients with metastatic melanoma. Approximately 50% of metastatic melanoma patients are diagnosed with brain metastases, while an additional 40% are noted to have brain metastases at autopsy (3).

Due to a paucity of reliable animal models with brain metastases, our understanding of the underlying mechanisms of brain metastases is limited. Metastasis is a complex multistep process that includes cell proliferation, invasion of basement membrane, intravasation into blood circulation, survival in blood stream, organ tropism, extravasation, and colonization into specific organs (4). At each step the cell interacts with its surroundings and is under constant survival pressure. A critical component in this process is the epithelial to mesenchymal transformation (EMT) (5). Similarly, when the metastatic cell exits the blood stream and enters the destination organ it again changes from mesenchymal to epithelial phenotype (MET). Multiple genetic and epigenetic factors play a role in EMT and MET, SMAD and non-SMAD signaling, MAP kinase pathway including BRAF alterations, and PI3K/AKT pathway (6–11).

## Blood Brain Barrier

The presence of the blood brain barrier (BBB) makes brain metastases unique compared to other sites of metastases. The BBB serves a protective role by restricting the movement of cellular components and solutes between systemic circulation and brain. It is comprised of endothelial cells with tight junctions on the systemic circulation side, and pericytes, astrocyte endfoot, and nerve endings on the neuronal side (12). Several efflux transporters of the ATP-binding cassette (ABC) gene family, such as the P-glycoprotein (P-gp) and breast cancer resistance protein (BCRP), are upregulated in the endothelial cells of the BBB. These transporters, in addition to being drug specific transporters, play a crucial role in the elimination of toxins and drugs from the CNS (13). While the endothelial barrier restricts the movement of cells across the BBB, it may paradoxically enable the transmigration of malignant cells during the process of diapedesis. The exact mechanism of BBB penetration is unknown however there is data to suggest extravasation of malignant cells which proliferate intravascularly, damage the vessels, and disrupt the BBB, thereby leading to metastases formation. Once the metastatic cells are intracranial, the protective BBB limits the immune surveillance and penetrance of systemic therapies (12). Data from Osswald et al. shows brain metastases can be effectively targeted by certain drugs that are designed to cross the BBB, specifically, small molecular inhibitors (14). Similarly, the blood-tumor barrier (BTB) significantly impacts the efficacy of therapeutic agents in brain metastases. This was clearly described by Lockman et al. (15) with an analysis of the variable permeability of brain metastases from breast cancer human and murine models, impeding the delivery of therapeutic drugs into metastases. Additionally, MRI contrast enhancement to electron-dense tracers demonstrates increased BTB permeability in some brain metastases (16, 17). Since measurements of drug levels in active brain metastases are difficult to obtain, data on the exact mechanism of the BTB is limited. Published results emphasize the need for molecularly targeted therapies with a higher potential for penetration of the BTB in order to reach therapeutic levels within tumors (18).

## Genomics of Brain Metastases

The advent of more efficient next-generation sequencing techniques have enhanced our understanding of genomic alterations in brain metastases. Whole exome analysis of brain metastases and matched primary tumor from 86 patients showed genomic heterogeneity and branched evolution (19). These results indicate that although metastatic sites share common genes with primary tumors, they develop unique genetic alterations, providing survival advantage in the brain (19). This study also revealed increased frequency of *PI3K/Akt, mTOR, CDK* alterations in brain metastases. Another relevant study analyzed 16 melanoma brain metastases and matched extracranial sites, with hotspot mutations, mRNA expression patterns, protein expression and activation, and copy number variations (20). The *PI3K/Akt* pathway was enriched in the brain metastases. Overall similarity was noted in most other driver mutations. A multicenter next generation sequencing and gene expression study of ~17,000 unmatched primary tumors from non-small cell lung cancer (NSCLC), breast cancer and melanoma demonstrated higher TOP2A expression in brain metastases (21). There was also increased expression of proteins critical in DNA synthesis and repair. This research provides important genetic information for future drug development in the treatment of brain metastases.

## ALK Fusions and Other Gene Fusions

Anaplastic lymphoma kinase (*ALK*) fusions were first noted in a subset of anaplastic large cell lymphoma (22) with translocations involving *ALK* on chromosome 2p and molecular partners such as *NPM-ALK, TPM3-ALK*, and *TFG-ALK* (23). *ALK* fusions occur in ~3% of non-small cell lung cancer (NSCLC). The identification of inversions of echinoderm microtubule-associated protein-like 4 (*EML4*) with *ALK* in Japanese women with lung cancer led to the development of drugs targeting *EML4-ALK* fusions (24). This aberrant fusion leads to activation of ALK kinase and downstream signaling pathways including the RAS–mitogen-activated protein kinase (MAPK), JAK-STAT and, phosphoinositide 3-kinase (PI3K) –AKT. One study estimates the incidence of brain metastases in ALK-fusion harboring NSCLC (ALK-NSCLC) to be >45% in 3 years (25). Several tyrosine kinase inhibitors (TKI) are developed for the management of ALK-NSCLC. Crizotinib, the first FDA approved TKI for ALK-NSCLC, has limited CNS penetration with a CSF serum ratio of < 0.1 to 0.26% (26, 27). The phase 3 clinical trial of crizotinib in ALK-NSCLC included 79 patients with previously treated and stable brain metastases, with 39 randomized to receive crizotinib while 40 received chemotherapy (28). The intracranial disease control rate at 12 weeks was 85% in patients treated with crizotinib compared to 45% in those treated with chemotherapy (*p* < 0.001) (29). A retrospective analysis of two randomized clinical trials of crizotinib in treatment naïve ALK-NSCLC showed that 20% of the patients who had extracranial disease progression, developed new brain metastases (30). In summary, although crizotinib provides better intracranial disease control, it has poor CNS penetration and 1 in 5 patients treated with crizotinib develop brain metastases.

The next generation of ALK inhibitors, including ceritinib and alectinib, have improved intracranial activity. The ASCEND-1, a phase 1 trial of ceritinib, included 124 patients with stable brain metastases (31). Data for measurable intracranial lesions was available for 14 patients, 10 of whom had prior exposure to an ALK inhibitor. Intracranial responses were reported in 7 patients and 3 had stable disease. In a phase 2 trial of ceritinib, 100 of 140 total patients had brain metastases, however, only 20 had measurable target lesions. The intracranial response rate was 45%, demonstrating good CNS activity (32).

Alectinib is another ALK inhibitor that has been studied in patients with brain metastases. A phase 3 Japanese study compared alectinib to crizotinib in ALK-NSCLC (J-ALEX study). In the analysis of 207 patients, 43 had brain metastases at enrollment. The 1-year cumulative incidence rates for intracranial progression was lower in alectinib group at 5.9% compared to 16.8% in the crizotinib group (33). Similar results were reported in the international phase 3 trial comparing alectinib to crizotinib (ALEX) in newly diagnosed metastatic ALK-NSCLC where 18 of the 152 patients (12%) in the alectinib group and 68 of the 151 patients (45%) had CNS progression at 18 months (34). All patients in the study had MRI brain at enrollment. The time to CNS progression was longer in patients' treatment with alectinib compared to crizotinib, additionally, 12-month cumulative incidence of brain metastases was 41.4% in the crizotinib group compared to 9.4% in the alectinib group. Data from two phase 2 studies of alectinib were pooled to evaluate the intracranial efficacy and included 136 patients with brain metastases from ALK-NSCLC who had progressed on crizotinib (35). The CNS disease response rate was 64%. In conclusion, alectinib has better CNS activity compared to crizotinib.

There is early clinical data to support intracranial activity with a new ALK inhibitor, brigatinib. Up to 70% of patients with crizotinib resistant ALK-NSCLC in the early phase clinical trials of brigatinib had brain metastases (36). In total, 59 patients had measurable brain metastases, and 31 (53%) of them had intracranial responses to brigatinib. Another exploratory analysis of two phase 2 clinical trials confirmed the intracranial activity of brigatinib (37). Phase 3 clinical trial with brigatinib are showing promising results (38).

ROS1 fusions are reported in 2% of advanced NSCLC (39). Approximately 20% of these patients have brain metastases at diagnosis (40). Crizotinib also has activity against ROS1 fusion, however, as mentioned earlier, it has limited intracranial penetration (41). Lorlatinib is a TKI with activity in ROS1 fusion NSCLC, and preliminary results from an ongoing phase 2 study indicate intracranial responses in 3 of 12 patients with brain metastases (42).

The TRK family of tyrosine kinases, TRKA, TRKB, and TRKC are encoded by *NTK* genes (43). NTK gene fusions lead to activation of the TRK receptors, which increase cell proliferation and survival by PI3K and Ras/MAPK/ERK pathways. Entrectinib is a TKI with activity against *ALK, ROS1*, and *NTRK* gene fusions. In a phase 1–2 clinical trial of entrectinib, 5 of the 8 patients with primary or metastatic disease to the brain demonstrated intracranial responses (44). Larotrectinib is another *NTK* fusion inhibitor in clinical development. Although this drug showed limited CNS penetration in preclinical studies, one patient with NSCLC in the phase I study had 18% reduction in the size of brain metastases (45).

LOXO-292 selectively targets RET and early studies show activity against activating *RET* fusions/mutations. Drilon et al. recently presented data from a phase I study of patients with *RET* fusion± malignancies including NSCLC, papillary thyroid cancer, and medullary thyroid cancer (42, 46). The NSCLC cohort included 3 patients with brain metastases with a significant reduction in the tumor burden suggesting activity in the brain. BLU-667 is another highly selective RET inhibitor which shows promise in patients with brain metastases (47).

## BRAF Mutation

V-raf murine sarcoma viral oncogenes homolog B1 (BRAF) is a potent activator of the mitogen-activated protein kinase (MAPK) signaling pathway. The RAS/BRAF/MAPK/ERK pathway, critical for cell survival and proliferation, is altered in ~30% of all malignancies (48). The *BRAF* gene mutation has been identified in melanoma, lung, colon, and thyroid cancers (49). In melanoma, *BRAF*^*V*600*E*^ mutation accounts for 90% of all *BRAF* mutations, while *BRAF*^*V*600*K*/*R*/*D*^ are less common (50).

Up to 50% of all advanced melanoma patients harbor *BRAF* mutations, making it a good target for BRAF inhibitors. Conservative estimates suggest that about 20% of *BRAF* mutant metastatic melanoma patients develop brain metastases (51).

Early studies with BRAF inhibitors show intracranial activity. An intracranial response rate of 16% was noted with single agent vemurafenib in unresectable brain metastases from metastatic melanoma (52). A phase I study of dabrafenib demonstrated intracranial responses in 9 of 10 melanoma patients (53) whereas a larger multicenter phase 2 study with single agent dabrafenib enrolled 172 patients with *BRAF*^*V*600*E*/*K*^ mutant melanoma with brain metastases (54). *BRAF* mutated patients had improved intracranial responses, with 40% (29 of 74) of treatment naïve and 30% (20 of 65) of previously treated brain metastases patients responding to single agent dabrafenib.

The combination of BRAF inhibitor and MEK inhibitor was found to be superior with less adverse effects in the treatment of advanced melanoma (55, 56). The combination of dabrafenib and trametinib was evaluated in BRAF mutated metastatic melanoma patients with brain metastases in the COMBI-MB trial (57). Patients were enrolled into four cohorts. Cohort A included patients with BRAF^V600E^ mutation who had good performance status, no symptoms from brain metastases and had not received intracranial therapy. BRAF^V600E^ patients who had good performance status, asymptomatic brain metastases with progression of intracranial metastases after initial local therapy were enrolled to cohort B. Cohort C had asymptomatic patients with a good performance status but had BRAF^V600D/K/R^ mutation. Finally, cohort D had patients with symptomatic brain metastases, from metastatic melanoma with BRAF^V600E/D/K/R^ mutation. The response rates in cohorts A through D were 58% (44 of 76 patients), 56% (9 of 16 patients), 44% (7 of 16 patients), and 59% (10 of 17 patients), respectively. These encouraging responses across all the cohorts make the combination of BRAF + MEK inhibitors a reasonable strategy in the management of patients with BRAF mutated metastatic melanoma with brain metastases. Studies with new BRAF and MEK inhibitor combinations will also provide more data (58, 59). BRAF directed therapy, dabrafenib plus trametinib is now approved for treatmentlung cancer patients where BRAF mutations are noted in about 2–4% of patients (60), however, their utility in lung cancer with brain metastases is yet to be evaluated.

## CDK Pathway Alterations

Cell cyclin dependent kinases (CDK4/6) play a role in transitioning cells from G1 to S phase of cell division. The phosphorylation of tumor suppressor proteins like retinoblastoma protein is a key function of CDK4/6 which leads to cell division and proliferation (61). *CDKN2A* alterations are common in hormone receptor positive breast cancer patients. Palbociclib, abemaciclib, and ribociclib are the three CDK inhibitors that are approved for management of hormone receptor positive advanced breast cancer. Whole exome analysis of matched brain metastases patients and primary tumors showed increased frequency of alterations which might sensitize brain metastases to CDK inhibitors (19). Currently, clinical trials with CDK inhibitors in patients with brain metastases are enrolling patients, including a phase 2 study of palbociclib in recurrent brain metastases (NCT 02896335).

## EGFR Mutation

Epidermal Growth Factor Receptor (EGFR) is a transmembrane protein of the Human Epidermal Receptor (HER) family. The HER family encompasses 4 different receptors namely: EGFR/erbB1/HER1, erbB2/HER2, erbB3/HER3, and erbB4/HER4 receptors. All these receptors have tyrosine kinase roles that activate signal transduction inducing cell proliferation. *EGFR* overexpression or mutations are common in NSCLC, head and neck cancer, and colon cancer.

EGFR targeted therapies have been successful in the treatment of advanced lung cancer. Gefitinib and erlotinib are the two first-generation EGFR-TKIs that have improved progression-free survival (PFS) in advanced EGFR-NSCLC patients (62, 63). In the pivotal studies leading to their FDA-approval, patients with brain metastases were excluded. Both gefitinib and erlotinib have CSF concentrations higher than inhibitory concentration *in vitro*, despite being substrates for efflux pumps (64, 65). More recently, studies have evaluated the efficacy of gefitinib and erlotinib in EGFR-NSCLC patients with brain metastases (66–68). For example, Wu et al. enrolled 48 NSCLC patients with intracranial progressive disease after initial platinum-based chemotherapy to receive erlotinib (67). Although patients were not enriched for *EGFR*, the intracranial PFS and overall survival (OS) was 10.1 and 18.9 months, respectively. With an aim of obtaining higher intracranial concentration for erlotinib, investigators tried higher pulse doses which show promising results (69–71). The combination of erlotinib and radiation therapy was evaluated in two studies (66, 68). In a phase 2 study, 40 patients with brain metastases from NSCLC were treated with erlotinib and WBRT (68). There was no increase in toxicity, and an impressive response rate of 60% was noted. The median OS was 11.8 months, and the median survival was 19.1 months in *EGFR* mutated patients. A larger phase 3 attempted to evaluate the efficacy of radiation therapy and erlotinib in NSCLC patients with 1–3 brain metastases. The study design included three groups: WBRT plus stereotactic radiosurgery (SRS), WBRT plus SRS plus temozolomide, and WBRT plus SRS plus erlotinib. The study did not meet accrual and was not enriched for EGFR mutant patients, and it did not show significant differences in OS. Significant toxicity was noted with the combination of WBRT plus erlotinib, with ~50% of the patients experiencing serious adverse effects, including myocardial ischemia and hemorrhagic stroke. Gefitnib is another first generation TKI has modest intracranial activity (72, 73). The intracranial activity of afatinib was reported in a case series of 100 patients with brain metastases where afatinib in a compassionate use program, however, the median time to intracranial progression was 3.9 months. Osimertinib is an EGFR inhibitor with activity against T790M, a mutation that confers resistance to first and second generation EGFR tyrosine kinase inhibitors. Pooled analysis from two phase 2 studies of 50 patients with measurable brain metastases showed intracranial response rates of 54%; 75% of patients at 9 months had an ongoing response (74). Most ongoing clinical trials with osimertinib have now allowed enrollment of patients with stable asymptomatic brain metastases. In the recently reported phase III clinical trial of upfront osimertinib, median intracranial PFS at 6 months was 87% in the osimertinib group compared to 71% in the standard EGFR-TKI group (75). This progression free survival benefit was sustained at 18 months. The CNS progression was lower in the osimertinib compared to standard EGFR-TKI (6 vs. 15%) (76). Osimertinib has activity in leptomeningeal disease as well (77). At the initial efficacy assessment of a phase 1 clinical trial of osimertinib in EGFR mutant NSCLC with leptomeningeal disease, 33% (7 of 21) of patients were responding to treatment (77).

## HER2 Alterations

HER2 receptor, a transmembrane EGFR receptor, with no known ligands for the HER2 receptor, is activated by homodimerization and heterodimerization (78). When activated, HER2 receptors lead to tumor growth, proliferation, and more invasiveness. The Ras/MAP kinase and PIK3/mTOR are the common downstream signaling pathways activated by HER2 overexpression and mutation. HER2 overexpression is primarily identified in 20% of breast cancer (79) but can be present in 30% of upper gastrointestinal malignancies like esophageal adenocarcinoma and gastro-esophageal junction carcinoma (80). HER2 overexpression generally indicates aggressive behavior (79). Several different strategies have been adopted to improve outcomes in these patients including monoclonal antibodies like trastuzumab, and pertuzumab, TKIs such as lapatinib, neratinib, tesevatinib, and the antibody drug conjugate trastuzumab-emtansine (T-DM1).

Trastuzumab was the first monoclonal antibody that showed improvement in OS in the metastatic, adjuvant, and neo-adjuvant setting (81, 82). However, a number of trastuzumab treated patients had intracranial disease recurrence. This is likely partly due to the inherent biology of HER2 overexpressing breast cancer, and partly because trastuzumab has poor penetration across the BBB (83, 84). The plasma-to-CSF concentration of trastuzumab has been evaluated in patients with brain metastases by using immunoenzymatic tests (85). Notably, intracranial trastuzumab levels can change dramatically with radiation therapy; prior to radiation therapy the CSF to plasma levels of trastuzumab were reported to be low (1:420), with an increase (1:79) after radiotherapy. Other studies with radio-labeled-trastuzumab have corroborated this finding (86, 87). Although some retrospective studies have shown improvement in OS patients with brain metastases treated with trastuzumab, it may be due to improved extracranial disease control (88, 89). Pertuzumab showed promising clinical activity when added to a regimen containing trastuzumab in various clinical settings (90, 91). Clinical evidence of CNS penetration of pertuzumab was demonstrated in a demonstrating prolongation of the interval from treatment to development of CNS metastases, which was 15.0 months in the pertuzumab-treated population compared to 11.9 months (92). Lapatinib is a small molecule TKI inhibiting EGFR and HER2 receptor activation. In the absence of CNS metastases, lapatinib has an intracranial concentration of 3%, which increases to 25% in the presence of brain metastases (93). This is change in intracranial concentration has been attributed to altered blood brain barrier by brain metastases. In a phase 2 study, 39 patients with HER2 overexpressing breast cancer and measurable brain metastases who progressed on trastuzumab were treated with lapatinib and results showed only one partial response (94). In a multicenter single arm study of lapatinib in combination with capecitabine (a nucleoside inhibitor), 29 of 45 patients (66%) had a partial response (95). The combination of lapatinib and topotecan (a topoisomerase I inhibitor) failed to improve response rates compared to lapatinib and capecitabine (96). A combination of lapatinib and cabazitaxel (a microtubule inhibitor) has also been safely combined in brain metastases patients and the results of the phase 2 study have not been published (97). Neratinib, a newer HER2 targeting TKI approved for adjuvant treatment of breast cancer patients with HER2 overexpression was evaluated in HER2 overexpressing breast cancer brain metastases, the majority of which had progressed after WBRT (98), with an overall response of only 8%. The combination of neratinib plus capecitabine was recently evaluated in a phase 2 clinical trial with encouraging preliminary results showing a 12-month survival of 63% in 39 patients. Tesevatinib is another TKI which has shown safety and preliminary efficacy in brain metastases from breast and lung cancer patients (99, 100).

The antibody drug conjugate trastuzumab-emtansine (T-DM1) is an approved second line treatment option for metastatic HER2 overexpressing tumors after trastuzumab (101). Patients with brain metastases treated in the registration trial had improved survival with T-DM1 compared to lapatinib plus capecitabine (102).

## Immunotherapy

Monoclonal antibodies targeting immune-checkpoints (CTLA-4 and PD-1/PDL-1) have revolutionized the management of several advanced malignancies, particularly melanoma and NSCLC. Initial studies with ipilimumab, a CTLA-4 antibody, in melanoma patients with brain metastases showed modest responses, which was largely impacted by use of dexamethasone (103). A recent open label, multiinstituitional phase 2 study evaluated the combination of ipilimumab and nivolumab (anti PD-1 antibody) in melanoma patients with asymptomatic untreated brain metastases (104). The primary endpoint for this study was intracranial benefit rate, defined by stable disease for 6 months, or response to treatment. Ninety four patients were enrolled in the trial, and the results were impressive with 57% patients meeting the primary end-point while 26% had complete response. Pembrolizumab is another anti PD-1 antibody which was studied in a single center phase 2 clinical trial of patients with brain metastases from melanoma or NSCLC (105). The melanoma arm accrued 23 patients and 6 of them had intracranial response with a median OS of 17 months (106). An interim analysis for 18 NSCLC patients reported an intracranial response rate of 33%. These studies provide early but encouraging evidence for intracranial activity with these agents. An important limitation for immunotherapy is the use of dexamethasone for symptomatic brain metastases, and during radiation therapy.

## VEGF (Vascular Endothelial Growth Factor) Pathway

Angiogenesis and neovascularization play a critical role in the development of brain metastases, thus anti-angiogenic therapy could be a promising strategy. Bevacizumab is a monoclonal antibody which has an established track record of anti-VEGF activity. Preliminary results from a phase 2 trial of the combination of bevacizumab and carboplatin in breast cancer patients with brain metastases showed a response rate of 45% (107). The favorable changes in MRI appearance is likely secondary to decreased inflammation from alteration of blood vessels by bevacizumab.

## Conclusion

The management of CNS metastatic disease remains challenging. Surgery and radiation are still the most common approaches to the management of brain metastases. The minimal progress in the management of brain metastases can be attributed to the unique challenges in drug delivery to the CNS, and the limited understanding of the genetic heterogeneity in brain metastases compared to primary tumors. Furthermore, most clinical trials have historically excluded patients with CNS disease. Our knowledge of the genetics of brain metastases is increasing and new targeted therapies with improved CNS penetration are in development. Finally, clinical trials dedicated to patients with brain metastases in all malignancies with an emphasis on translational science will provide insight and therapeutic options for this patient population. Table 1 provides a summary of clinical trials with targeted agents for brain metastases.

**Table 1:** Summary of selected studies of targeted therapies in brain metastases.

**Targeted therapy**	**Primary malignancy**	**Study design**	**Number of patients with brain metastases**	**Outcomes**
**ALK DIRECTED THERAPY**				
Crizotinib (29)	NSCLC	Subgroup analysis of a phase 3 trial	• 79 patients with stable BM • 39 treated with crizotinib • 40 treated with standard chemotherapy	12 week DCR 85% in the crizotinib group compared to 45% in the chemotherapy group
Ceritinib (32)	NSCLC	Subgroup analysis of a phase 2 trial	• 100 patients had asymptomatic BM • 20 had measurable BM	IC-RR: 45%
Alectinib (35)	NSCLC	Pooled analysis of two phase 2 trials	136 patients with BM who had progressed on crizotinib	IC-RR: 64%
Brigatinib (38)	NSCLC	Subgroup analysis of phase 2 trial	40 patients in the brigatinib group and 41 patients in the crizotinib had brain metastases	IC-RR: 78% in the brigatinib group compared to 29% in the crizotinib group
**BRAF-MEK DIRECTED THERAPY**				
Vemurafenib (52)	Melanoma	Phase 2 trial	• 90 patients with previously untreated BM	IC-RR: 18%
Dabrafenib (54)	Melanoma	Phase 2 trial	• 172 patients with BRAF mutant melanoma and BM	IC-RR of 40% in treatment naïve and 30% in previously treated patients
Dabrafenib and trametinib (57)	Melanoma	Multicenter, multicohort phase 2 trial	• Cohort A: 76 patients with BRAF^V600E^ mutation, good PS, asymptomatic and newly diagnosed BM • Cohort B: 16 patients with BRAF^V600E^, good PS, asymptomatic but progressive BM • Cohort C: 16 patients, asymptomatic, good PS, BRAF^V600D/K/R^ • Cohort D: 17 patients, symptomatic, BRAF^V600E/D/K/R^	IC-RR in Cohort A: 58% IC-RR in Cohort B: 56% IC-RR in Cohort C: 44% IC-RR in Cohort D: 59%
**EGFR DIRECTED THERAPY**				
Erlotinib (67)	NSCLC	Phase 2 trial	• 48 patients with progressive BM	IC-PFS: 10.1 months
Erlotinib (68)	NSCLC	Phase 2 trial	• 40 patients with progressive BM, concurrent with radiation	IC-RR: 60%
Erlotinib (66)	NSCLC	Phase 3 trial	• 41 patients treated with WBRT/SRS plus erlotinib	MST: 6.1 months 6 month IC-DCR: 10%
Osimertinib (75)	NSCLC	Phase 3 trial	• 61 patients treated with osimertinib 67 patients treated with standard EGFR-TKI	PFS at 6 months: 87% vs. 71%. PFS at 18 months: 58% vs. 40%
**HER2 DIRECTED THERAPY**				
Lapatinib plus capecitabine (95)	Breast cancer	Phase 2 trial	• 45 patients with BM	IC-RR: 66%
Neratinib plus capecitabine (98)	Breast cancer	Phase 2 trial	• 39 patients with BM	12 month OS: 63%
**IMMUNOTHERAPY**				
Ipilimumab plus nivolumab (104)	Melanoma	Phase 2 trial	• 94 patients with BM	IC benefit: 57%
Pembrolizumab (105, 106)	Melanoma NSCLC	Phase 2 trial	• 23 patients with BM 18 patients with BM	IC-RR: 26% IC-RR: 33%

*NSCLC, Non-small cell lung cancer; BM, brain metastases; DCR, disease control rate; IC-RR, Intracranial response rate; MST, median survival time; OS, overall survival; PFS, progression free survival; IC benefit, 6 month stable disease, complete or partial response*.

## Future Directions

A multi-disciplinary approach including primary medical oncologists, radiation oncologists, neuro-oncologists, and neurosurgeons is critical in the management patients with brain metastases. Histology and molecular profiling should guide treatment options. For specific malignancies such as melanoma and NSCLC, immune checkpoint inhibitiors have durable responses with and without radiation or surgery. Furthermore, patients with targetable driver mutations can be treated with novel systemic targeted agents with better CNS penetration than previously used chemotherapy. Dedicated clinical trials, brain metastases consortiums and a personalized approach to this patient population will focus on many remaining unanswered questions.

## Author Contributions

VV, JC, and PB were involved in the initial planning, writing, and editing the manuscript.

### Conflict of Interest Statement

PB has received speaker's honorarium from Genentech, and Merck and is a consultant for Lilly, Tesaro, Angiochem and Genentech-Roche. PB has also received grant support to MGH from Merck, BMS and Pfizer. VV received honorarium from Elsevier. JC is a consultant for Sanofi-Genzyme.
